# Reduced Fertilization and Magnesium Supplementation: Modulating Fruit Quality in Honey Pomelo (*Citrus maxima* (Burm.) Merr.)

**DOI:** 10.3390/plants13192757

**Published:** 2024-10-01

**Authors:** Da Su, Yunfei Jiang, Biao Song, Zhaozheng Wu, Xiaojun Yan, Zhiyuan He, Delian Ye, Jie Ou, Yingzhe Zeng, Liangquan Wu

**Affiliations:** 1 Key Laboratory of Genetics, Breeding and Multiple Utilization of Crops, Ministry of Education, Key Laboratory of Biological Breeding for Fujian and Taiwan Crops, Ministry of Agriculture and Rural Affairs, College of Agriculture, Fujian Agriculture and Forestry University, Fuzhou 350002, China; ydl2015@fafu.edu.cn; 2 International Magnesium Institute, College of Resources and Environment, Fujian Agriculture and Forestry University, Fuzhou 350002, China; song53362024@163.com (B.S.); 16565710@fafu.edu.cn (X.Y.); hzy13593020593@163.com (Z.H.); 5220831082@fafu.edu.cn (J.O.); liangquan01@163.com (L.W.); 3 Department of Plant, Food and Environmental Sciences, Faculty of Agriculture, Dalhousie University, 50 Pictou Road, Truro, NS B2N 5E3, Canada; yunfei.jiang@dal.ca; 4 Pinghe Bureau of Agriculture and Rural Affairs, Zhangzhou 363700, China

**Keywords:** *Citrus maxima* (Burm.) Merr., fruit quality, phenolic, flavonoid, phytic acid, mineral nutrition

## Abstract

The excessive use of chemical fertilizers in the Guanxi honey pomelo production area has led to severe soil acidification and magnesium (Mg) deficiency, adversely affecting pomelo fruit quality. To address this issue, an integrated nutrient optimization model crucial for ensuring the sustainable and environmentally friendly development of the Guanxi honey pomelo industry has been explored. In a three-year experiment, two fertilizer treatments were implemented: a farmer fertilizer practice (FP) and an NPK reduction plus foliar Mg fertilizer (OPT + fMg). We investigated the impact of this integrated optimized fertilization measure on pomelo fruit quality from three aspects: flavor (sugars and organic acids), nutrition (vitamin C and mineral elements), and antioxidant properties (phenolics, flavonoids, and phytic acid). The results revealed that the OPT + fMg treatment improved fruit flavor by reducing acidity (titratable acid, citric acid, and quinine), while having a minimal impact on sugar components (sucrose, fructose, and glucose). Additionally, the OPT + fMg treatment increased the total phenolics, total flavonoids, and phytic acid in the fruit peel, enhancing its potential antioxidant quality. However, the OPT + fMg treatment reduced the mineral nutrient quality (excluding calcium) in the fruit. As for the fruit developmental period, the OPT + fMg treatment significantly increased the total flavonoid concentration in the peel from the mid-expansion fruit stage, followed by notable increases in phytic acid in the peel during the mid-to-late expansion fruit stage. The total phenolic concentration in the peel significantly rose only during the late fruit development stage. The most pronounced effect was observed on phytic acid in both peel and pulp. The influence of the OPT + fMg treatment on the mineral nutrients (excluding calcium) primarily occurred during the mid-to-late expansion fruit stage. Overall, the OPT + fMg treatment significantly improved the comprehensive nutritional quality of pomelo fruit, providing valuable insights for scientifically reducing fertilizer application while enhancing fruit quality.

## 1. Introduction

Pomelo, belonging to the Rutaceae family, is a prominent member of the citrus genus, recognized for its high yield and large fruit size. As the world’s largest producer of pomelos, China boasts a rich variety of pomelo germplasm resources, contributing to nearly half of the global production (FAOSTAT, 2019) [1]. Pinghe County in Fujian Province is renowned as the origin and primary cultivation area of the Guanxi honey pomelo (*Citrus maxima* (Burm.) Merr.). In 2021, the annual production of honey pomelos in Pinghe County reached 2.105 million tons, marking an increase of nearly 16% compared to 2018 [2]. Pinghe County has thus become the leading area in China for the production and export of high-quality pomelos. Historically, the yield-increasing model dominated by chemical fertilizers has led to the excessive input of fertilizers, particularly nitrogen (N), phosphorus (P), and potassium (K) in citrus orchards. For instance, the average annual N fertilizer input in Chinese citrus orchards is as high as 500 kg ha^−1^, which is 2.5–3.3 times that of the high-yield and efficient citrus growing regions in countries like the United States and Brazil [2,3,4,5]. Chen et al. (2022) revealed that significant reductions in traditional fertilizer applications, by nearly 53%, were particularly achievable in areas like Pinghe County [6].

Excessive fertilizer application significantly reduces fertilizer use efficiency. Our previous results from a ^15^N-urea labeling study in the Pinghe orchard showed that the N use efficiency of the farmer fertilizer practice was only 8.37%. However, after optimizing the fertilization strategy by reducing N by 80%, the N use efficiency increased to 23.66%, a nearly threefold increase. Furthermore, the long-term reduction of an NPK application in the NPK-surplus orchards of Pinghe County did not significantly reduce honey pomelo yield [6]. Excessive fertilizer application, especially ammonium N fertilizer, exacerbated soil acidification in southern China due to the microbial oxidation of NH_4_^+^ producing large amounts of H^+^ [7,8]. Over the past thirty years, the overall soil pH in Pinghe County’s orchards has decreased from 5.7 to 4.3. A comprehensive evaluation of 319 typical orchard soils in Pinghe County by Li et al. revealed an average soil pH of only 4.34 [9]. Soil acidification not only affects the effective utilization of mineral nutrients and organic matter but weakens the ion exchange capacity of the soil, directly leading to nutrient imbalances, such as magnesium (Mg) and calcium (Ca) deficiencies. Due to the high rainfall in southern China, soil Mg leaching is more prominent than Ca, resulting in a more significant deficiency of soil available Mg in citrus orchards [2,10,11]. Surveys in southern China showed that nutrient imbalances in citrus orchard soils were a common issue, with 73% of orchard soils and 93% of citrus leaves exhibiting varying degrees of Mg deficiency (<60 mg kg^−1^) [10,12]. Similarly, in Pinghe County, 35.6% of the pomelo leaves exhibited chlorosis due to Mg deficiency, which was found in 77.4% of the soils [9].

Currently, within the entire citrus production system, including the honey pomelo, farmers primarily focus on a heavy NPK application, with limited awareness of the need for a timely Mg supplementation. Our previous work comprehensively evaluated the ecological (e.g., carbon emissions) and economic effects of fertilizer reduction and Mg supplementation in pomelo orchards. By reducing fertilizer application by nearly 86% from the farmer fertilizer practice, production costs can be reduced by 35%, and the annual carbon emissions and carbon footprint can be decreased by 90%. These significant environmental benefits were achieved without sacrificing honey pomelo yield [2]. However, the impact of an integrated nutrient optimization strategy of fertilizer reduction and Mg supplementation on the comprehensive quality of pomelos has not been intensively studied. The Guanxi honey pomelo is rich in bioactive compounds (such as phenolics, flavonoids, and organic acids) and nutrients (minerals) [13,14]. These bioactive and nutritional components are abundant in both fresh pulp and processed peel products [15]. These bioactive compounds mainly protect human health by eliminating oxygen free radicals and enhancing antioxidant capacity (e.g., anti-cancer, inhibiting chronic inflammation, and boosting immunity) [16]. Previous studies on bioactive compounds (such as phenolics and flavonoids) mainly focused on the isolation and identification of the bioactive components, varietal and genotypic differences [13], and their relationship with antioxidant properties, with limited research on the effects of fertilization management and the nutrient regulation on these components [17].

Based on our previous farmer surveys and an exploration of the nutrient demand patterns of Guanxi honey pomelo trees, we identified two obstacles in most pomelo orchards in Pinghe: excessive fertilizer application and commonly occurring Mg deficiency. This experiment proposed an integrated nutrient optimization model that reduced the NPK application by nearly 80% while supplementing the Mg fertilizer through a foliar application. This study aimed to explore the impact of this integrated fertilization model on the comprehensive quality of honey pomelos (e.g., flavor quality, nutritional quality, and potential antioxidant quality). We hypothesized that improving nutrient input in pomelo orchards would enhance the overall quality of honey pomelo fruits. Our study may provide insights for scientifically reducing fertilizer application to achieve better quality control and promote the green development of the honey pomelo industry.

## 2. Results

### 2.1. Effect of Fertilizer Reduction and Mg Supplementation on the Flavor Quality of Mature Pomelo Fruits

**Figure 1:**
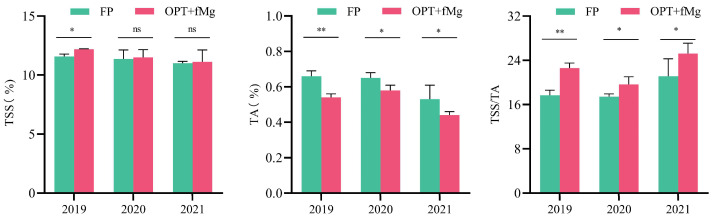
The effect of the two fertilization treatments on total soluble solids (TSS), titratable acidity (TA), and their ratio (TSS/TA) in pomelo (*Citrus maxima* (Burm.) Merr.) fruit at the ripening stage (2019–2021). Note: 1. * and ** represent a significant difference at *p* < 0.05 and *p* < 0.01 under the two fertilization treatments, respectively; ns indicates not significant. 2. The horizontal axis represents different years. 3. FP: farmer fertilizer practice; OPT + fMg: NPK reduction plus foliar Mg fertilizer. 4. TSS: total soluble solids; TA: titratable acid; TSS/TA: the ratio of TSS to TA.

The reduction of fertilizers combined with Mg supplementation (OPT + fMg) improved the flavor characteristics of mature pomelo fruits (Figure 1). Results over three consecutive years (2019–2021) indicated that the OPT + fMg treatment significantly increased the TSS (total soluble solids)/TA (titratable acid) of pomelo fruits, resulting in a more balanced sweet and sour taste, and notably enhanced fruit flavor. The increase in the TSS/TA was primarily due to a decrease in the TA rather than an increase in the TSS. Additionally, over the years, there has been a trend of a further reduction in fruit acidity and an increase in the TSS/TA.

### 2.2. Effect of Fertilizer Reduction and Mg Supplementation on Bioactive Compounds (Total Phenolic, Total Flavonoid, and Phytic Acid) in Pomelo Fruits at Different Developmental Stages

**Figure 2:**
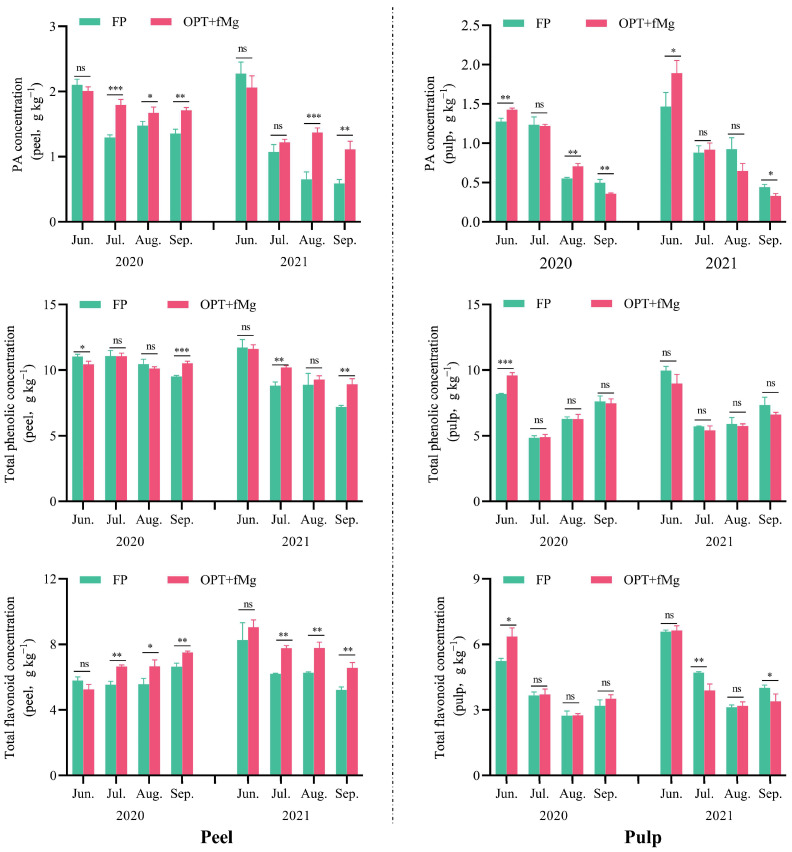
The effect of the two fertilization treatments on phytic acid, total phenolic, and total flavonoid concentrations at various growth stages of pomelo (*Citrus maxima* (Burm.) Merr.) fruit (left: peel; right: pulp). Note: 1. *, **, and *** represent a significant difference at *p* < 0.05, *p* < 0.01 and *p* < 0.001 under the two fertilization treatments, respectively; ns indicates not significant. 2. The horizontal axis represents different years (2020 and 2021) and various growth stages (Jun, Jul, Aug, Sep) of pomelo fruits. 3. FP: farmer fertilizer practice; OPT + fMg: NPK reduction plus foliar Mg fertilizer. 4. PA: phytic acid.

The OPT + fMg treatment significantly affected the concentration of bioactive compounds in pomelo fruits. This impact varied depending on the fruit parts (peel and pulp), developmental stages (early expansion, mid-expansion, late expansion, and ripening), and different bioactive compounds (total phenolics, total flavonoids, and phytic acid) (Figure 2).

Peel: Compared to the farmer fertilizer practice, the OPT + fMg treatment significantly increased the total phenolic, total flavonoid, and phytic acid concentrations in the peel at the ripening stage. However, the degree of influence and the primary periods of impact on different bioactive components varied (Figure 2, left). The OPT + fMg treatment started to increase the total flavonoid concentration in the peel from the mid-expansion stage (July) and continued until the ripening stage. At the ripening stage, the total flavonoid concentration in the peel significantly increased compared to the farmer fertilizer practice. The OPT + fMg treatment notably increased the phytic acid concentration in the peel starting from the mid to late expansion stages, with an increase of 25.9% (2020) and 88.0% (2021) at the ripening stage compared to the farmer fertilizer practice. The total phenolic concentration in the peel significantly increased mainly in the late fruit development stage, with an increase of 10.5% (2020) and 24.1% (2021) at the ripening stage compared to the farmer fertilizer practice. Overall, the OPT + fMg treatment significantly increased the concentrations of all three bioactive compounds in the peel, with the largest increase observed for phytic acid. Among the different years, the increase was most pronounced in 2021.

Pulp: Compared to the farmer fertilizer practice, the OPT + fMg treatment did not significantly affect the total phenolic and total flavonoid concentrations in the pulp at different developmental stages (except in the early expansion stage, 2022) (Figure 2, right). 

Unlike the effects on the total phenolic and total flavonoid concentrations, the OPT + fMg treatment significantly reduced the phytic acid concentration in the pulp during the ripening stage, primarily due to inhibitory effects in the late fruit development stage. However, the OPT + fMg treatment significantly increased phytic acid concentration in the pulp during the early expansion stage.

### 2.3. Effect of Fertilizer Reduction and Mg Supplementation on Mineral Element Concentrations (P, Ca, Mg, Zn, and Fe) in Pomelo Fruits at Different Developmental Stages

The OPT + fMg treatment significantly affected the concentrations of mineral elements in pomelo fruits. The impact of the OPT + fMg treatment on different mineral elements (P, Ca, Mg, Zn, Fe) varied depending on the fruit parts (peel and pulp) and developmental stages.

Peel: The changes in the mineral element concentrations of the mature peel showed consistent trends across the different years (Table 1). Specifically, compared to the farmer fertilizer practice, the OPT + fMg treatment significantly reduced the concentrations of P, Mg, Zn, and Fe in the mature peel (except for Mg in 2021). Overall, the reductions in the concentrations of trace elements like Zn and Fe were greater than those of P and Mg. The Ca concentration in the mature peel did not show significant differences between the two fertilizer treatments. The degree of influence on different mineral elements also varied with the developmental stage of the peel. Combined results from the two years indicated that the treatment mainly started significantly reducing the Fe concentration in the peel from the mid-expansion stage (except for the late expansion stage in 2020), while the reduction in P and Zn concentrations was most significant during the mid to late fruit development stages. The treatment did not significantly affect the Mg concentration in the peel at different developmental stages (except for the mature stage in 2020). 

Pulp: Combined results from the two years (Table 2) showed that, compared to the farmer fertilizer practice, the OPT + fMg treatment significantly reduced the concentrations of P, Zn, and Fe in the mature pulp only in 2021. The Mg concentration in the mature pulp did not show significant changes, while the Ca concentration in the mature pulp increased significantly, with an increase of 9.92% in 2020 and 10.34% in 2021. The OPT + fMg treatment mainly significantly increased the Ca concentration in the pulp during the early (early expansion) and late (mature) fruit development stages. Although the treatment significantly reduced the Mg concentration in the pulp during the mid-expansion stage, there were no significant changes in the Mg concentration in the mature pulp between the different treatments. The treatment only significantly reduced the P concentration in the pulp during the late fruit development stage (2021). It significantly reduced the Zn concentration in the pulp during the mid and late expansion stages and significantly reduced the Fe concentration in the pulp during the early fruit development stage. 

### 2.4. Effect of Fertilizer Reduction and Mg Supplementation on Sugar, Organic Acid Components, and Vitamin C Concentrations in Mature Pomelo Fruits

**Figure 3:**
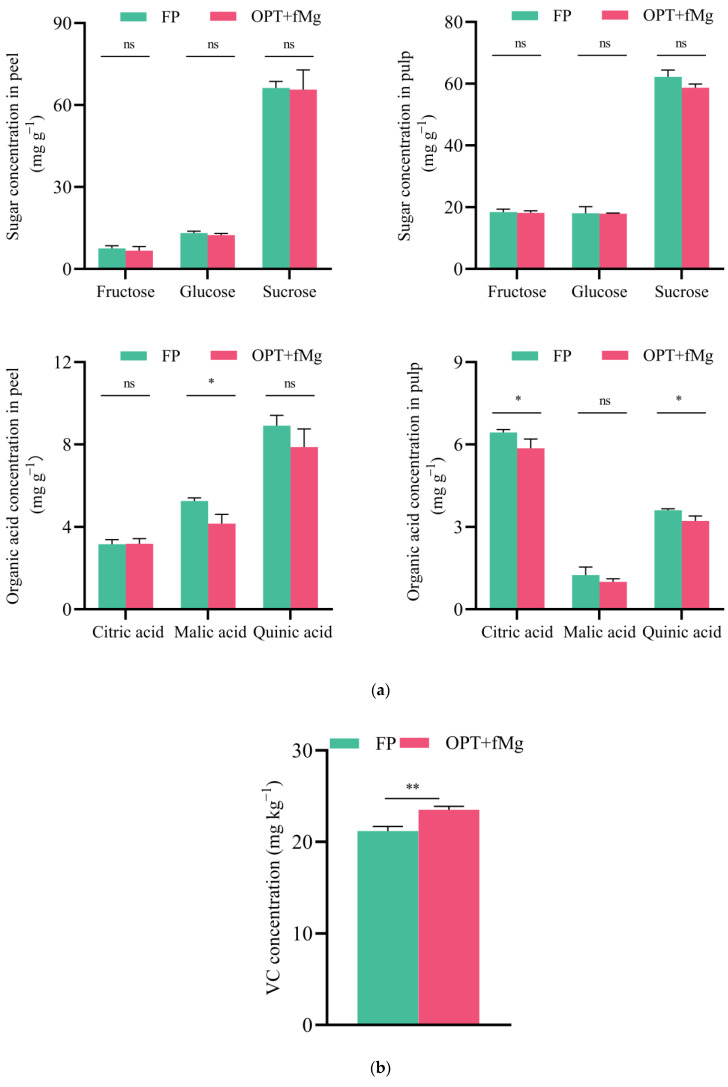
The effect of the two fertilization treatments on fruit (peel and pulp), sugar (sucrose, glucose, fructose), organic acids (citric acid, malic acid, quinic acid) (**a**), and pulp vitamin C concentrations (**b**) of pomelo (*Citrus maxima* (Burm.) Merr.) fruit at the ripening stage in 2021. Note: 1. * and ** represent a significant difference at *p* < 0.05 and *p* < 0.01 under the two fertilization treatments, respectively; ns indicates not significant. 2. FP: farmer fertilizer practice; OPT + fMg: NPK reduction plus foliar Mg fertilizer. 3. VC means vitamin C.

The OPT + fMg treatment significantly affected the organic acid components (citric acid, malic acid, and quinic acid) and vitamin C (VC) concentration in mature pomelo fruits (both peel and pulp) but had no significant impact on the sugar components (sucrose, glucose, and fructose) (Figure 3).

Sugars: The composition of sugar substances in Guanxi honey pomelo fruits showed significant differences between the fruit parts (peel and pulp). In the peel, sucrose had the highest proportion (76–77%), followed by glucose (15%), and fructose had the lowest proportion (8%). In the pulp, sucrose still had the highest proportion (61–63%), but the proportions of fructose and glucose were close (18–19%). The OPT + fMg treatment did not significantly affect the sugar concentration in either the peel or pulp.

Organic Acids: The composition of organic acids in pomelo fruits also showed significant differences between the fruit parts. In the peel, quinic acid had the highest proportion (51%), followed by malic acid (27–30%), and citric acid had the lowest proportion (18–21%). In the pulp, citric acid had the highest proportion (56–58%), followed by quinic acid (32%), and malic acid had the lowest proportion (10–11%). The OPT + fMg treatment had inconsistent effects on the organic acid components in the peel and pulp. In the peel, the OPT + fMg treatment significantly reduced the malic acid concentration, while changes in the quinic acid and citric acid were not significant. In the pulp, the treatment mainly reduced the concentrations of the two relatively high-proportion organic acids (citric acid and quinic acid) but had no significant effect on the malic acid concentration.

Vitamin C: The OPT + fMg treatment also significantly increased the VC concentration in the pulp, with an increase of 11%.

### 2.5. Effect of Fertilizer Reduction and Mg Supplementation on Mg Concentration in Different Functional Leaves of Pomelo

**Figure 4:**
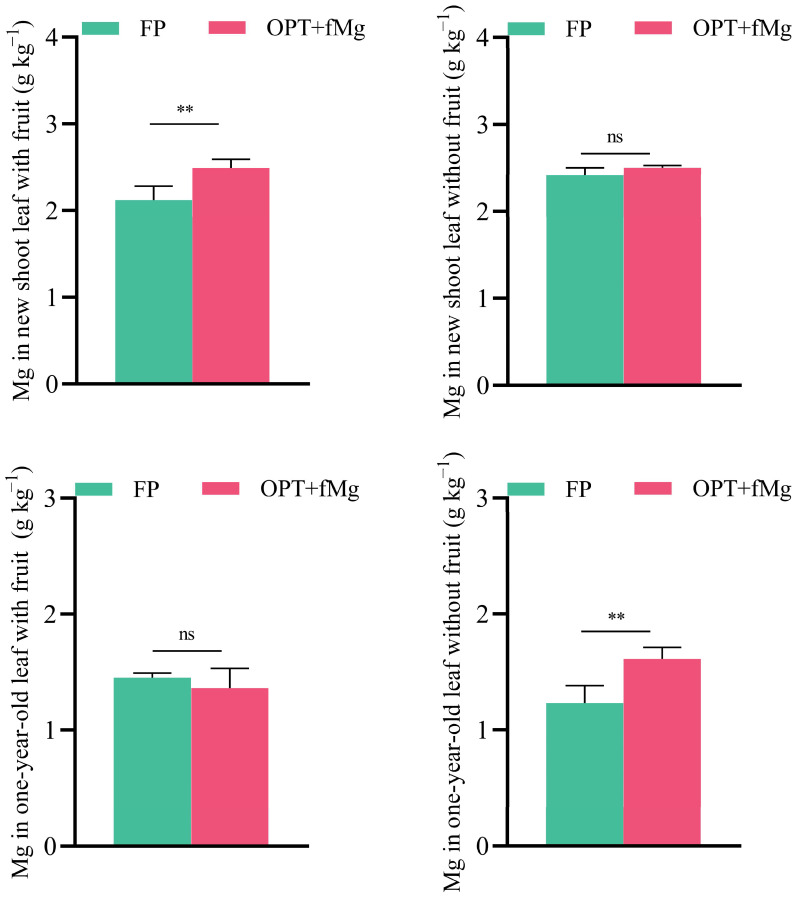
The effect of the two fertilization treatments on the Mg concentration in different leaves of pomelo (*Citrus maxima* (Burm.) Merr.) trees in 2019. Note: 1. ** represent a significant difference at *p* < 0.01 under the two fertilization treatments, respectively; ns indicates not significant. 2. FP: farmer fertilizer practice; OPT + fMg: NPK reduction plus foliar Mg fertilizer.

As shown in Figure 4, the effect of the OPT + fMg treatment on the Mg concentration in different functional leaves of pomelo varied based on the position and age of the leaves (e.g., new shoot leaf with fruit; new shoot leaf without fruit; one-year-old leaf with fruit; one-year-old leaf without fruit). The OPT + fMg treatment primarily increased the Mg concentration in new shoot leaf with fruit and one-year-old leaf without fruit. However, it did not significantly affect the Mg concentration in new shoot leaf without fruit and one-year-old leaf with fruit.

## 3. Discussion

In China, agricultural production is predominantly led by smallholder farmers, where the overuse of fertilizers has led to environmental concerns, low nutrient use efficiency, and quality deterioration. This phenomenon is particularly prominent in economic crop regions. Similar to other citrus-producing areas, Pinghe County, the main production area and origin of the Guanxi honey pomelo, faces the problem of excessive fertilizer input. This has resulted in nutrient surpluses (NPK) and deficiencies (Mg) in the soil, exacerbating nutrient imbalance. Soil surveys in Pinghe County revealed that 82.1% of pomelo orchards had a P surplus, and 77.4% had an exchangeable Mg deficiency [9]. To ensure the green and sustainable development of the Guanxi honey pomelo industry in Pinghe County, it is crucial to reduce the NPK inputs with the timely supplement of Mg [12].

### 3.1. The Impact of Excess Fertilizer Input on Citrus Quality and the Benefits of Mg Supplementation 

Nutrient balance is essential for ensuring high crop quality. Excess nutrients directly degrade fruit quality. In a previous study, high N levels significantly reduced soluble sugars in pineapple [18]. In another study, the TA was reduced, and the fruit flavor of apples was improved by reducing excessive K [19]. In a study of pumpkins, high NPK levels decreased the antioxidant concentration by 13–79% at various fruit development stages [20]. Moreover, the common issue of Mg deficiency in citrus orchards needs attention [12]. Both the soil and foliar application of Mg fertilizers can significantly reduce TA in fruits like the pomelo [6]. In this study, the effect of OPT + fMg (80% reduction in NPK + 4% MgSO_4_·7H_2_O) on the TSS concentration of the pomelo fruit was generally not significant, except in 2019. The coefficient of variation (CV) for TSS under both fertilization treatments remained relatively low across different experimental years. In addition, while the pomelo, like other citrus fruits, exhibited differences in the proportions of various sugar components (sucrose, glucose, and fructose) between the peel and pulp, the OPT + fMg treatment did not significantly affect the total sugar, sucrose, glucose, and fructose concentration in either the peel or pulp. However, the OPT + fMg treatment markedly reduced the TA concentration in the pulp, which, combined with changes in the organic acid composition, enhanced the fruit’s flavor. 

Fruit organic acids, including various secondary metabolites, play a crucial role in determining the fruit flavor by balancing sweetness and acidity. Previous studies on different citrus fruits have shown that citric acid dominates the organic acid profile in most mature citrus fruits, while malic acid and quinic acid are present in relatively lower proportions. Their levels and proportions vary widely among different citrus species and cultivars [21,22,23,24]. Guo et al. (2020) further found significant variations in the levels and proportions of organic acids across different fruit growth stages [21]. Specifically, quinic acid predominates during the first 50 days of fruit development, but decreases in later stages, whereas citric acid is initially low and increases at maturity. Consistent with findings from previous studies on other citrus fruits [21], our results for Guanxi honey pomelo pulp also showed that citric acid was the predominant organic acid. However, the quinic acid concentration in mature pulp was higher than that of malic acid, differing from findings in lime and lemon fruits, which may be related to the varietal characteristics of Sanhong honey pomelo used in this study. Moreover, significant differences were observed between the peel and pulp in the present study. Sun et al. (2012) analyzed the organic acid profile in the flavedo of pomelos, showing that the proportion of the three major organic acids was highest for quinic acid, followed by malic acid and citric acid [24]. This is consistent with our results in the peel of the pomelo. Further analysis demonstrated that the OPT + fMg treatment not only lowered TA but reduced the total organic acid concentration and its major constituents’ concentrations (Figure 3). It should be noted that the inhibitory effect of the OPT + fMg treatment on organic acid components varied between the peel and pulp. Specifically, it significantly decreased the peel malic acid concentration, which is the median component in terms of abundance, while it had a pronounced inhibitory effect on the more abundant citric and quinic acids in the pulp (Figure 3). Overall, the treatment had a greater impact on organic acids in the pulp, with the combined changes in sugars and acids ultimately determining the flavor profile of the fruit at maturity (e.g., the balance between sweetness and acidity). These findings suggest that the enhanced fruit TSS/TA ratio under the OPT + fMg treatment was primarily due to the significant reduction in acidity (TA, organic acids, and their components) rather than an increase in sweetness (Figure 1 and Figure 3). Excessive fertilizer application may disrupt the balance of carbon–nitrogen metabolism, inhibit photosynthetic capacity in leaves, and increase cellular oxidative damage. In this study, as nutrient input was further optimized, pomelo trees prioritized carbon and nutrient resources for basic metabolism (such as sugar synthesis) and growth. Since sugars are precursors for organic acid synthesis, this may explain the observed impact on organic acid and intermediate metabolite synthesis, leading to reduced acidity. Additionally, considering the common occurrence of Mg deficiency in pomelo orchard soils, and the necessity of Mg supplementation before visible symptoms of deficiency appear (such as chlorosis between leaf veins on older and mid-level leaves), this study utilized foliar Mg fertilization as a more efficient method compared to soil application [2]. This approach effectively coordinated and compensated for the potential effects of reduced NPK levels on leaf photosynthesis, sugar transport, and allocation [25]. The dual role of NPK fertilizer reduction and Mg supplementation in promoting sugar metabolism may be one of the factors contributing to the reduction in fruit acidity and the significant improvement in the sugar/acid ratio observed in this study.

A comprehensive evaluation of pomelo quality should consider both basic and unique fruit qualities. Citrus fruits, including the pomelo, are typically rich in vitamin C (VC) and phenolic compounds [15], offering significant nutritional and pharmacological benefits in addition to their distinctive flavors. Organic acids like citric, malic, and quinic acids are crucial for flavor balance and participate in antioxidant processes along with vitamin C, phenolics, and flavonoids. The impact of Mg on crop quality varies, but most studies indicate that Mg enhances the vitamin C concentration [26,27]. Consistent with previous findings in both conventional and organic orchards [28], this study demonstrated that, although the foliar Mg application did not increase the fruit Mg concentration on a dry matter basis (Table 2 and Table 3), the leaf Mg concentration of pomelo trees significantly increased (Figure 4). Additionally, we observed a notable alleviation of leaf chlorosis in the pomelo orchard. Therefore, Mg likely improved leaf chlorosis and enhanced photosynthetic efficiency, ultimately resulting in a significant 11% increase in vitamin C concentration in pomelo pulp following the OPT + fMg treatment (Figure 3). Phenolic compounds, particularly flavonoids, are secondary metabolites that affect fruit color, flavor, and aroma. They play crucial roles in scavenging free radicals and mitigating oxidative stress, thereby contributing to human health by improving the immune function, reducing inflammation, and addressing chronic conditions such as obesity, hypertension, diabetes, cancer, and cardiovascular diseases [29,30]. In addition to phenolic acids, phytic acid also exhibits bioactive properties and can act in conjunction with phenolics and flavonoids to provide antioxidant functions. Due to its multi-hydroxy biochemical structure, phytic acid chelates essential mineral cations such as Zn, Fe, Ca, and Mg, which are beneficial to human health, ultimately forming phytic acid salts (phytate) that are not absorbable by the human digestive system. This significantly reduces the effective absorption of these minerals in the body [31]. Consequently, previous research on phytic acid primarily focused on its anti-nutritional properties in cereal crops but neglected its potential antioxidant role in fruit. This study demonstrated that the phytic acid concentration in pomelo fruit peels was notably higher, with approximately three times the amount found in the pulp (Figure 2). Considering that the concentrations of total phenolic and total flavonoid were also higher in the peel compared to the pulp (Figure 2), and that the peel serves as a major raw material for deep processing industries, the bioactive compounds in pomelo peel can significantly enhance the value of processed products. Effective extraction techniques can capture these bioactive compounds, adding value to the final products.

### 3.2. Influence of Environmental Factors on the Accumulation of Phenolics, Flavonoids, and Phytic Acid

The synthesis and accumulation of phenolics, flavonoids, and phytic acid in horticultural plants are significantly affected by environmental factors. Growth stages, fruit types/varieties, fruit parts, climate conditions (such as light, temperature, soil nutrients), stress, hormones (like salicylic acid), and global warming all play critical roles in modulating the levels of these bioactive compounds [32,33,34,35]. Anttonen et al. [36] found that even minor changes in agricultural practices (such as fertilizer management, mulch color, and planting times) and environmental factors (such as light exposure and planting regions) can significantly alter the phenolic concentration and antioxidant activity in strawberries. Genotype, developmental stages, and environmental factors (light and temperature) also significantly influence the transcription of genes associated with flavonoid synthesis and the expression of related enzyme activities in fruits like strawberries, thereby affecting the synthesis and accumulation of flavonoids [37]. Among these environmental factors, fertilizer management is one of the most direct, significant, and controllable agronomic measures for regulating crop yield and quality. Studies on certain fruits have shown that reducing the N application can lead to an increase in phenolic concentration. Lower N levels improved the phenolics in grapevines and enhanced non-anthocyanin phenolics in dry red wine [38]. Organic fertilization with reduced N also positively increased polyphenolic antioxidant compounds in tomatoes [39]. Oloyede et al. [20] reported that, in pumpkins, increasing levels of NPK fertilizers (0–250 kg ha^−1^) resulted in a continuous and significant decrease in total phenolic and flavonoid concentrations. These findings suggest that the precise management of N fertilization can be an effective strategy to enhance bioactive compounds, such as phenolics, in horticultural crops, which may benefit human health. High N levels may primarily inhibit the synthesis of phenolic compounds by suppressing key enzymes (such as phenylalanine ammonia-lyase) and critical regulatory genes, significantly reducing the synthesis and accumulation of phenolics and their derivatives. Mg nutrition also significantly influences phenolic formation. Metabolomic studies have also shown that Mg may play a crucial role in influencing the metabolic expression of phenolic compounds and flavonoids in various plant species. Studies in apple peel showed that the appropriate Mg application in Red Fuji apple trees significantly increased anthocyanin biosynthesis-related genes and transcription factors, indicating a possible impact on the phenolic compound and flavonoid expression [40]. Consistent with previous results, this experiment demonstrated that a combined approach of reducing chemical fertilizer application and supplementing with Mg fertilizer significantly increased the total phenolic and total flavonoid concentrations in pomelo fruits (Figure 2). Given that phenolics and flavonoids are typical carbon-based compounds, based on the carbon/nitrogen balance theory, the regulation measures that promote carbon metabolism (such as increasing Mg fertilizer) and reduce excessive N input (chemical fertilizer reduction) might play a dual role in promoting the synthesis of phenolics. This could be one of the reasons why the combined approach of fertilizer reduction and Mg supplementation significantly increased the total phenolic and total flavonoid concentrations in the fruits in the present experiment. Furthermore, our results indicated that the positive regulation effect of the OPT + fMg treatment on the total phenolic and total flavonoid concentrations in the peel was more pronounced, possibly due to the higher concentrations of these compounds in the peel (Figure 2). Since the pomelo peel constitutes about 50% of the total fruit weight [41], the OPT + fMg treatment could play a positive role in enhancing the nutritional value and utilization of pomelo peel. After the OPT + fMg treatment, the phytic acid concentration in the peel, which was already high at maturity, increased further, while the phytic acid concentration in the pulp decreased (Figure 2). This suggests that the OPT + fMg treatment may enhance the deep processing of the entire fruit, including the extraction, utilization, and development of bioactive compounds. Due to the high overall P deposit in the citrus orchard soil [42], no additional P fertilizer was applied. The OPT + fMg treatment was based on the annual P nutrient utilization pattern of pomelo trees, ensuring normal growth and development. Moreover, P in cereal grains mainly exists in the form of phytic acid-P, whereas the P components in leaves and fruits are not [43]. Therefore, exogenous P or a P fertilizer practice may not be the primary factor affecting fruit phytic acid accumulation. Previous studies in rice have shown that exogenous sucrose significantly regulated the expression of the key phytic acid synthesis gene MIPS (the rate-limiting enzyme gene for phytic acid synthesis) [44]. Considering that Mg also plays a role in modulating the activity of key photosynthetic enzymes [45], and that carbohydrate accumulation mainly occurs in the pulp, Mg may influence sugar metabolism and ultimately reduce phytic acid concentration in the pulp. The metabolism of phytic acid in fruit peel and pulp may exhibit distinct regulatory mechanisms, and the physiological mechanisms by which nutrient inputs regulate phytic acid synthesis in the peel and pulp remain unclear and require further investigation. Our results further revealed that the regulatory effects of the OPT + fMg treatment on various quality traits differed depending on the fruit’s developmental stage and parts (peel and pulp). The flavonoid concentration in the peel began to increase significantly during the mid-expansion stage, whereas the phytic acid concentration in the peel started to rise significantly during the late-expansion stage, and the total phenolic concentration in the peel only increased significantly in the late fruit development stage. However, the impact of the OPT + fMg treatment on phytic acid in the pulp was observed at the early and late stages of fruit development, showing opposite trends (increasing at the early stage but decreasing at the late stage). This suggests that the regulation of bioactive components (such as phenolics, flavonoids, and phytic acid) in fruits by the OPT + fMg treatment should also consider the developmental stages and parts of the fruit to maximize the utilization and development of these bioactive components.

### 3.3. Impact on Mineral Elements and Quality Traits in Citrus Fruits

Citrus fruits, including pomelos, are rich in mineral elements such as K and trace elements like iron (Fe), making them a valuable part of healthy diets like the Mediterranean diet, which is why they are often recommended for individuals suffering from anemia and hemoglobin synthesis disorders caused by Fe deficiency [14]. In this study, the OPT + fMg treatment led to a significant decrease in the concentration of mineral elements (P, Mg, Zn, Fe) in the peel, except for Ca, with the impact on trace elements like Zn and Fe being more pronounced (Table 2 and Table 3). Since traditional NPK fertilizers contain small amounts of trace elements, reducing NPK fertilizers simultaneously results in a significant decrease in both major and trace elements in the peel. However, the Ca concentration in the peel did not decrease, and the Ca concentration in the pulp significantly increased. Previous research on tea plants showed that excessive N input led to soil acidification, significantly reducing exchangeable base cations like Ca^2+^ [46]. Overuse of P also tended to precipitate with soil Ca^2+^, particularly in subtropical acidic soils [47]. In this study, with an 80% reduction in N fertilizer and no additional P fertilizer, the lower NPK application may improve soil nutrient balance and health, enhancing Ca availability and facilitating its absorption by plants. Ca not only helps maintain the structural integrity of cell walls and the hardness of the peel, but is involved in the translocation of organic compounds (such as sugars and carbohydrates) from the leaves or tree body to the fruit, contributing to the synthesis and accumulation of sugars in the fruit [2]. This can enhance the quality of pomelos during the maturation stage and promote the preservation of the fruit during its shelf life. 

It is noteworthy that although the Mg concentration in mature fruits did not significantly increase, the analysis of Mg concentration in pomelo leaves showed that the OPT + fMg treatment increased the Mg concentration in the leaves, particularly in new shoot leaf with fruit and one-year-old leaf without fruit (Figure 4). This indicated that a foliar Mg fertilizer application promoted Mg storage in the tree and its translocation during fruit development, which may be one of the reasons why the OPT + fMg treatment enhanced the sugar/acid ratio and improved the flavor quality of the fruits. 

## 4. Materials and Methods

### 4.1. Experimental Site and Materials

The experiment was conducted over three years from 2019 to 2021 at the long-term experimental site at the Guanxi Honey Pomelo Science and Technology Backyard (STB) in Pinghe County, Fujian Province (24°02′ N, 24°35′ W). The site has a subtropical monsoon climate, with a soil pH ranging from 4.3 to 4.4. The test material was 10-year-old Sanhong honey pomelo trees, which are widely cultivated and economically significant in this region, representing one of the most typical Guanxi honey pomelo varieties.

### 4.2. Experimental Design

Previous experiments have explored the impacts of different levels of fertilizer reduction and Mg supplementation (soil application and foliar spraying) on the yield of the honey pomelo and found two limiting factors in Pinghe orchards: excessive fertilizer input and insufficient soil available Mg supply [1,2,6]. Compared with the farmer’s fertilizer practice, both an 80% reduction in the NPK and Mg fertilizer (MgO) input improved the basic flavor quality of the fruit while maintaining or slightly increasing the yield [6]. Therefore, this experiment further combined these two known nutrient optimization measures. Specifically, to optimize fertilization (80% reduction in NPK), a foliar application of 4% MgSO_4_·7H_2_O, OPT + fMg, was applied to explore the impact of this integrated nutrient optimization model on the comprehensive quality of the honey pomelo compared to the farmer fertilizer practice. It is noteworthy that, due to the high P concentration in Pinghe County soils, with an annual surplus of approximately 450 kg ha^−1^, which is 14.5 times the amount needed for the normal growth and development of pomelo trees [48], no additional P fertilizer was provided in this experiment. The specific input amounts of N, P, K, and Mg fertilizers in the two treatments are shown in Table 3. The application periods for N, P, and K fertilizers in the orchards were December (30%), February (spring sprout fertilizer, 20%), April (fruit setting fertilizer, 30%), and June (fruit expansion fertilizer, 20%). The source of the foliar-applied Mg was MgSO_4_·7H_2_O (4%), with an application rate of 7 L per tree, applied in March (1/3), April (1/3), and May (1/3). Each fertilizer treatment was set up in three plots, and each plot contained 12 trees. Except for the differences in fertilizer input between the two treatments, all other cultivation and field management measures were consistent throughout the growth period. 

**Table 3:** The amount of fertilizer used in both the farmer fertilizer practice (FP) and an NPK reduction plus foliar Mg fertilizer (OPT + fMg).

Fertilizer Source	FP	OPT + fMg
N (kg ha^−1^): Urea (46% N)	1084	230
P (kg ha^−1^): Diammonium phosphate (18% N, 46% P_2_O_5_)	914	0
K (kg ha^−1^): Potassium sulphate (50% K_2_O)	906	230
Mg: MgSO_4_·7H_2_O (4%)	0	7 L/tree

Note: FP: farmer fertilizer practice; OPT + fMg: NPK reduction plus foliar Mg fertilizer.

### 4.3. Sampling Methods

During the ripening period (September) of each year from 2019 to 2021, pomelo fruit samples were collected to analyze the basic flavor quality (TSS, TA, and TSS/TA). From 2020 to 2021, fruit samples were collected at typical stages of fruit development, including the early expansion stage (June), mid-expansion stages (July and August), and the ripening stage (September). The harvest/ripening stage typically occurs within a week before the Mid-Autumn Festival (the 15th day of the 8th month in the Chinese lunar calendar). These samples were analyzed for bioactive components (phytic acid, total phenolic, and total flavonoid, different fruit growth stages in 2020–2021), and mineral nutrients (P, Ca, Mg, Zn, and Fe, different fruit growth stages in 2020–2021) in both the pulp and peel of the pomelo fruit. In addition, in 2019, samples of various functional leaves—new shoot leaf with fruit (NLF), new shoot leaf without fruit (NL), one-year-old leaf with fruit (OLF), and one-year-old leaf without fruit (OL)—were collected during the ripening period to analyze leaf Mg concentration. In the ripening period of 2021, fruit samples were also selected to analyze sugar components (fructose, glucose, and sucrose) and organic acid components (citric acid, malic acid, and quinic acid) in both peel and pulp, as well as pulp vitamin C (VC) concentrations. Within each plot, fruit trees with similar growth vigor were randomly selected, and samples were taken from a fixed position (consistently chosen from the upper-middle part of the outer canopy). The various functional leaves (2019) corresponded to the sampled pomelos. The selected pomelo fruits were uniform in terms of growth and maturity, size, appearance, and peel color, and were free from damage or pest infestation. All samples (leaves and fruits) were taken to the laboratory immediately. The samples were quickly washed with deionized water, dried with paper towels, and cut into small pieces. The peel, pulp, and functional leaves used for analyzing bioactive components (phytic acid, total phenolic, and total flavonoid) and mineral elements (P, Ca, Mg, Zn, and Fe) were oven-dried until a constant weight was achieved. The dried samples were then crushed to a fine homogeneous powder and stored at −20 °C until analysis. Fresh samples were used for analyzing TSS, TA, and TSS/TA immediately after harvest. To analyze the sugar components (fructose, glucose, and sucrose) and organic acid components (citric acid, malic acid, and quinic acid), the fresh fruit tissues were separated into peel and pulp, then lyophilized, ground in liquid nitrogen, and stored at −20 °C until analysis [21]. In each treatment, nine representative trees were selected from three plots. The fruits and leaves with different functions from each plot were pooled together as one replicate. Specifically, each tree provided two fruits and one corresponding functional leaf.

### 4.4. Analyses of Fruit Quality 

For basic flavor qualities, TSS (%) was assessed using a digital refractometer (PAL-1; Atago Co., Ltd., Tokyo, Japan). TA (%) was measured using a phenolphthalein indicator and NaOH titration technique. The sugar/acid ratio was calculated as the TSS/TA ratio. Vitamin C (VC) concentration (mg kg^−1^) was determined using 2,6-dichlorophenol indophenol sodium. The concentration of sugars (glucose, fructose, and sucrose) and organic acids (citric acid, malic acid, and quinic acid) (mg g^−1^) were analyzed using HPLC, as described by Albertini et al. (2006) [21]. For bioactive components, the total phenolic concentration was quantified using spectrophotometry with the Folin–Ciocalteu reagent, following the procedure described by Tawaha et al. (2007) [49], and expressed as gallic acid equivalents (GAE) in g per kg of dry material. The total flavonoid concentration was determined using the method of Chen et al. (2020) and expressed as g of rutin equivalents (RE) per kg of dry material [50]. PA levels (g kg^−1^) were measured spectrophotometrically as described by Liang et al. (2008) [51]. The mineral element concentrations (P, Ca, Mg, Zn, Fe) in both the leaves and fruit (peel and pulp) were analyzed using ICP-OES [3].

### 4.5. Statistical Analysis

Data were processed using Microsoft Office Excel 2019 and presented as the mean ± standard deviation. Statistical analysis was conducted using IBM SPSS Statistics 21. The differences between the two fertilization strategies were evaluated using an independent *t*-test.

## 5. Conclusions

The OPT + fMg treatment significantly improved fruit flavor and bioactive components but reduced fruit mineral quality. This improvement was primarily achieved by lowering the fruit’s acidity (TA and the major organic acids in the pulp: citric acid and quinic acid) rather than increasing its sweetness (soluble solids and fruit sugar components: sucrose, fructose, and glucose), thereby enhancing the flavor quality of the fruit. Additionally, the increase in bioactive components (such as total phenolics, total flavonoids, and phytic acid) in the peel contributed to improving the potential antioxidant quality of the fruit. The increased pulp vitamin C (VC) concentration also elevated the fruit’s nutritional quality. However, due to the reduction of chemical fertilizers, the concentrations of mineral elements in the fruit (both peel and pulp), except for Ca, significantly decreased, with a more pronounced effect on the peel. Furthermore, the influence of the OPT + fMg treatment on various quality traits varied with the fruit’s developmental stage. The impact on bioactive components in the peel and mineral nutrition (except for Ca) in the fruit was concentrated in the mid to late stages of fruit development. 

## Figures and Tables

**Table 1:** The effect of the different fertilization treatments on peel mineral (P, Ca, Mg, Zn, Fe) concentrations at various growth stages of pomelo (*Citrus maxima* (Burm.) Merr.) fruit.

		2020 (Year)			2021 (Year)		
		FT	OPT + fMg	Fluctuating Rate (%)	FT	OPT + fMg	Fluctuating Rate (%)
P (g kg^−1^)	Jun.	1.19 ± 0.07	1.17 ± 0.03	−1.68 ^ns^	0.87 ± 0.06	1.03 ± 0.09	18.39 ^ns^
	Jul.	0.81 ± 0	0.75 ± 0.03	−7.41 *	0.80 ± 0.02	0.82 ± 0.03	2.50 ^ns^
	Aug.	0.65 ± 0.02	0.65 ± 0.01	0 ^ns^	0.72 ± 0.03	0.68 ± 0.02	−5.56 ^ns^
	Sep.	0.66 ± 0.02	0.53 ± 0.01	−19.70 ***	0.80 ± 0.02	0.61 ± 0.04	−23.75 **
Ca (g kg^−1^)	Jun.	7.97 ± 0.28	6.93 ± 0.33	−13.05 *	3.66 ± 0.12	5.57 ± 0.18	52.19 ***
	Jul.	5.60 ± 0.04	6.35 ± 0.36	13.39 *	3.86 ± 0.19	5.29 ± 0.31	37.05 **
	Aug.	6.08 ± 0.16	6.62 ± 0.11	8.88 **	3.56 ± 0.36	6.13 ± 0.22	72.19 ***
	Sep.	7.14 ± 0.23	6.81 ± 0.03	−4.62 ^ns^	4.92 ± 0.71	5.51 ± 0.41	11.99 ^ns^
Mg (g kg^−1^)	Jun.	1.68 ± 0.02	1.62 ± 0.03	−3.57 ^ns^	1.46 ± 0.02	1.45 ± 0.01	−0.68 ^ns^
	Jul.	1.64 ± 0.06	1.59 ± 0	−3.05 ^ns^	1.33 ± 0.04	1.39 ± 0.07	4.51 ^ns^
	Aug.	1.51 ± 0.03	1.46 ± 0.03	−3.31 ^ns^	1.21 ± 0.03	1.20 ± 0.03	−0.83 ^ns^
	Sep.	1.53 ± 0.04	1.42 ± 0.03	−7.19 *	1.45 ± 0.09	1.33 ± 0.05	−8.28 ^ns^
Zn (mg kg^−1^)	Jun.	21.86 ± 1.53	9.63 ± 0.98	−55.95 ***	15.33 ± 1.54	20.65 ± 3.46	34.70 ^ns^
	Jul.	16.58 ± 1.26	14.01 ± 2.70	−15.50 ^ns^	14.44 ± 2.13	11.86 ± 1.81	−17.87 ^ns^
	Aug.	17.54 ± 2.63	9.59 ± 2.09	−45.32 *	13.1 ± 1.85	10.43 ± 0.99	−20.38 ^ns^
	Sep.	20.36 ± 2.42	10.41 ± 0.87	−48.87 **	13.54 ± 1.85	9.55 ± 0.82	−29.47 *
Fe (mg kg^−1^)	Jun.	19.04 ± 1.87	11.98 ± 1.71	−37.08 **	16.31 ± 1.87	19.08 ± 2.14	16.98 ^ns^
	Jul.	19.35 ± 2.03	6.73 ± 0.38	−65.22 ***	19.13 ± 3.01	10.44 ± 0.85	−45.43 **
	Aug.	12.91 ± 3.30	9.29 ± 2.19	−28.04 ^ns^	19.01 ± 2.55	7.05 ± 0.74	−62.91 **
	Sep.	15.11 ± 2.72	4.53 ± 1.18	−70.02 **	15.83 ± 2.39	9.62 ± 1.76	−39.23 *

Note: 1. *, **, and *** represent a significant difference at *p* < 0.05, *p* < 0.01 and *p* < 0.001 under the two fertilization treatments, respectively; ns indicates not significant. 2. FP: farmer fertilizer practice; OPT + fMg: NPK reduction plus foliar Mg fertilizer.

**Table 2:** The effect of the different fertilization treatments on pulp mineral (P, Ca, Mg, Zn, Fe) concentrations at various growth stages of pomelo (*Citrus maxima* (Burm.) Merr.) fruit.

		2020 (Year)			2021(Year)		
		FT	OPT + fMg	Fluctuating Rate (%)	FT	OPT + fMg	Fluctuating Rate (%)
P (g kg^−1^)	Jun.	2.31 ± 0.03	2.39 ± 0.11	3.46 ^ns^	2.37 ± 0.11	2.28 ± 0.05	−3.80 ^ns^
	Jul.	1.53 ± 0.03	1.45 ± 0.04	−5.23 ^ns^	1.58 ± 0.02	1.52 ± 0.02	−3.80 *
	Aug.	1.26 ± 0.01	1.28 ± 0.03	1.59 ^ns^	1.54 ± 0.07	1.41 ± 0.03	−8.44 ^ns^
	Sep.	1.24 ± 0.06	1.18 ± 0.03	−4.84 ^ns^	1.68 ± 0.10	1.47 ± 0.04	−12.5 *
Ca (g kg^−1^)	Jun.	3.43 ± 0.11	3.69 ± 0.08	7.58 *	1.59 ± 0.29	2.28 ± 0.09	43.40 *
	Jul.	1.97 ± 0.04	1.89 ± 0.07	−4.06 ^ns^	1.23 ± 0.04	1.23 ± 0.13	0 ^ns^
	Aug.	1.49 ± 0.10	1.58 ± 0.08	6.04 ^ns^	0.76 ± 0.08	1.32 ± 0.15	73.68 **
	Sep.	1.21 ± 0.01	1.33 ± 0.06	9.92 *	0.87 ± 0.01	0.96 ± 0.03	10.34 **
Mg (g kg^−1^)	Jun.	1.44 ± 0.02	1.47 ± 0.09	2.08 ^ns^	1.06 ± 0.10	1.24 ± 0.04	16.98 ^ns^
	Jul.	1.02 ± 0.02	0.93 ± 0.02	−8.82 **	0.85 ± 0.02	0.81 ± 0.02	−4.71 *
	Aug.	0.84 ± 0.04	0.80 ± 0.04	−4.76 ^ns^	0.72 ± 0.02	0.73 ± 0.03	1.39 ^ns^
	Sep.	0.70 ± 0.04	0.73 ± 0.02	4.29 ^ns^	0.69 ± 0.02	0.74 ± 0.04	7.25 ^ns^
Zn (mg kg^−1^)	Jun.	23.77 ± 1.40	19.4 ± 2.17	−18.38 *	15.62 ± 1.07	15.94 ± 2.14	2.05 ^ns^
	Jul.	16.3 ± 1.49	12.19 ± 1.89	−25.21 *	15.47 ± 2.09	10.96 ± 1.04	−29.15 *
	Aug.	16.56 ± 2.50	9.53 ± 1.93	−42.45 *	15.29 ± 1.02	9.69 ± 1.42	−36.63 **
	Sep.	14.20 ± 2.1847	11.83 ± 1.82	−16.69 ^ns^	13.81 ± 0.98	10.54 ± 1.61	−23.68 *
Fe (mg kg^−1^)	Jun.	17.78 ± 1.39	8.83 ± 1.16	−50.34 **	14.51 ± 2.8	6.18 ± 1.35	−57.41 **
	Jul.	15.05 ± 0.85	12.57 ± 1.35	−16.48 ^ns^	11.11 ± 1.11	9.9 ± 1.76	−10.89 ^ns^
	Aug.	14.17 ± 1.56	10.84 ± 1.32	−23.50 *	13.43 ± 1.47	12.48 ± 0.75	−7.07 ^ns^
	Sep.	10.16 ± 1.19	10.12 ± 1.11	−0.39 ^ns^	14.77 ± 3.4	5.96 ± 0.65	−59.65 *

Note: 1. * and ** represent a significant difference at *p* < 0.05 and *p* < 0.01 under the two fertilization treatments, respectively; ns indicates not significant. 2. FP: farmer fertilizer practice; OPT + fMg: NPK reduction plus foliar Mg fertilizer.

## Data Availability

The raw data supporting the conclusions of this article will be made available by the authors on request.
